# Gene-Based Diagnosis of Tuberculosis from Oral Swabs with a New Generation Pathogen Enrichment Technique

**DOI:** 10.1128/spectrum.00207-22

**Published:** 2022-05-19

**Authors:** Young Ae Kang, Bonhan Koo, Ock-Hwa Kim, Joung Ha Park, Ho Cheol Kim, Hyo Joo Lee, Myoung Gyu Kim, Youngwon Jang, Na Hyun Kim, Yong Seo Koo, Yong Shin, Sei Won Lee, Sung-Han Kim

**Affiliations:** a Department of Internal Medicine, Division of Pulmonary and Critical Care Medicine, College of Medicine, Yonsei University, Seoul, Republic of Korea; b Department of Internal Medicine, Division of Pulmonary and Critical Care Medicine, Severance Hospital, Seoul, Republic of Korea; c Institute of Immunology and Immunological Disease, College of Medicine, Yonsei University, Seoul, Republic of Korea; d Department of Biotechnology, College of Life Science and Biotechnology, Yonsei University, Seoul, Republic of Korea; e Department of Pulmonary and Critical Care Medicine, Asan Medical Center, University of Ulsan College of Medicine, Seoul, Republic of Korea; f Division of Pulmonology and Critical Care Medicine, Department of Internal Medicine, Chungnam National University Sejong Hospital, Sejong, Republic of Korea; g Department of Infectious Diseases, Asan Medical Center, University of Ulsan College of Medicine, Seoul, Republic of Korea; h Department of Neurology, Asan Medical Center, Seoul, Republic of Korea; Indian Institute of Science Bangalore

**Keywords:** tuberculosis, rapid diagnosis, oral swab, gene-based diagnosis

## Abstract

A rapid and sensitive diagnosis is crucial for the management of tuberculosis (TB). A simple and label-free approach via homobifunctional imidoesters with a microfluidic platform (SLIM) assay showed a higher sensitivity than the Xpert MTB/RIF assay in the diagnosis of pulmonary TB (PTB). Here, we evaluated the efficacy of the SLIM assay for oral swab samples from cases of suspected PTB. Patients with clinically suspected PTB were prospectively enrolled and oral swab samples were processed using the SLIM assay and the attending physicians were blinded to the results of the SLIM assay. TB cases were defined as those treated with anti-TB chemotherapy for at least 6 months at the discretion of the specialists based on their clinical features and conventional laboratory results, including the Xpert assay. A total of 272 patients (with TB, *n *= 128 [47.1%]; without TB, *n *= 144 [52.9%]; mean age, 59.8 years) were enrolled. Overall, the sensitivity of the oral swab-based SLIM assay (65.6%) was higher than that of the sputum-based Xpert assay (43.4%; *P* = 0.001). Specifically, the SLIM oral swab assay showed a notably higher sensitivity in culture-negative TB cases compared with the Xpert assay (69.0% [95% CI: 49.2 to 84.7%] versus 7.4% [95% CI: 0.9 to 24.3%]; *P* = 0.001). The specificity of the SLIM and the Xpert assays was 86.1% (95% CI: 79.3 to 91.3%) and 100% (95% CI: 97.2 to 100%), respectively. When only culture-confirmed cases were analyzed, the SLIM oral swab was comparable to sputum Xpert in sensitivity (64.7% versus 54.3%, *P* = 0.26). The oral swab-based SLIM assay showed a superior sensitivity for TB diagnosis over the sputum-based Xpert assay, especially for culture-negative cases.

**IMPORTANCE** The development of a rapid, accessible, and highly sensitive diagnostic tool is a major challenge in the control and management of tuberculosis. Gene-based diagnostics is recommended for the rapid diagnosis of pulmonary tuberculosis (PTB), but its sensitivity, such as Xpert MTB/RIF assay (Xpert), drops in cases with a low bacterial load. It can only be applied to sputum samples, and it is quite difficult for some patients to produce an adequate amount of sputum. We evaluated the clinical validity of an oral swab-based microfluidic system, i.e., the SLIM assay. The SLIM assay showed a significantly higher sensitivity than the Xpert assay, especially in smear-negative TB cases. This non-sputum-based SLIM assay can be a useful diagnostic test by overcoming the limitations of conventional sputum-based tests in pulmonary TB.

## INTRODUCTION

Tuberculosis (TB) caused by infection with Mycobacterium tuberculosis (MTB) still ranks as a leading cause of death worldwide (1). Rapid and accurate diagnosis of pulmonary tuberculosis (PTB) in its early stage is vital for the successful control of the transmission of TB and for improving the treatment outcomes. In 2011, the World Health Organization (WHO) endorsed the use of the Xpert MTB/RIF assay (Xpert; Cepheid, Sunnyvale, CA, USA), a novel, rapid, automated, cartridge-based real-time PCR (PCR) method (2), for initial diagnosis of patients suspected of active PTB (3). In the landmark study, the Xpert assay showed a high sensitivity of 98.2% in acid-fast bacilli (AFB) smear-positive TB cases. However, the sensitivity was as low as 72.5% in smear-negative cases (4), and data from real-world settings reported a sensitivity of only around 60% to 74% (5, 6). Indeed, the sensitivity of Xpert for TB detection is inadequate when only a few bacilli are present in a clinical specimen.

The more recently developed Xpert-MTB/RIF Ultra assay showed a superior sensitivity to the Xpert assay (63% versus 46%) for diagnosing smear-negative PTB (7), but it was still not high enough. In addition, the Xpert assays can only be applied to sputum samples, which are occasionally hard to acquire from young children and asymptomatic patients with paucibacillary diseases. Furthermore, sputum collection is prone to producing potentially infectious aerosols that present a hazard for health care workers and fellow patients (8).

To overcome these limitations of the current TB diagnostics, we have developed a new assay for diagnosing PTB that involves simple and rapid pathogen enrichment by homobifunctional imidoesters (HIs) using a microfluidic system followed by conventional MTB PCR, i.e., the SLIM assay (9). The SLIM assay showed significantly better performance over the Xpert assay in terms of sensitivity (60%; 95% confidence interval [CI]: [47% to 72%] versus 37% [95% CI: 25% to 50%], *P* = 0.001) in the diagnosis of pulmonary TB (PTB) using sputum samples without a significant decrease in specificity (10).

To expand the clinical applicability of the SLIM assay, we investigated its performance for the diagnosis of PTB from oral swab samples. Specifically, this real-world, practice-based study was performed in patients with clinically suspected PTB in a country with an intermediate burden of TB and a low burden of the human immunodeficiency virus (HIV).

## RESULTS

### Participants.

A total of 272 patients suspected of PTB were enrolled in this study. The patients’ mean age was 58.8 ± 15.2 years and 174 (64.0%) were male. Malignant diseases (34.6%) and diabetes mellitus (19.9%) were the most common underlying diseases, followed by transplant status (4.8%) and liver cirrhosis (4.4%). Only one (0.4%) patient had an HIV infection (Table 1). A total of 52 patients (19.9%) had a history of previous pulmonary TB. Cough was the most common symptom (25%) Almost half of the participants (49.3%) had no specific respiratory symptoms and only had radiographic abnormalities.

**TABLE 1:** Baseline characteristics of the participants

Characteristic	Total	Treated as TB	Not TB	*P* value
	(*n *= 272)	(*n *= 128)	(*n *= 144)	
Age, yrs ± SD^*a*^	58.8 ± 15.2	56.4 ± 16.0	60.9 ± 14.1	0.014
Male sex, *n* (%)	174 (64.0)	85 (66.4)	89 (61.8)	0.43
Symptoms, *n* (%)				
Cough	68 (25.0)	29 (22.7)	39 (27.1)	0.4
Sputum	47 (17.3)	17 (13.3)	30 (20.8)	0.1
Hemoptysis	12 (4.4)	4 (3.1)	8 (5.6)	0.33
Fever	28 (10.3)	12 (9.4)	16 (11.1)	0.64
Night sweat	6 (2.2)	2 (1.6)	4 (2.8)	0.69
Dyspnea	15 (5.5)	7 (5.5)	8 (5.6)	0.98
Chest pain	8 (2.9)	7 (5.5)	9 (6.3)	0.78
General weakness	16 (5.9)	2 (1.6)	6 (4.2)	0.29
Chest radiograph abnormality, only	134 (49.3)	56 (43.8)	78 (54.2)	0.09
Previous TB history, *n* (%)	52 (19.9)	21 (16.4)	31 (21.5)	0.28
AFB smear positive, *n* (%)	46 (16.9)	35 (27.3)	11 (7.6)	<0.0001
Mycobacterial culture, TB isolated, *n* (%)	93 (34.2)	93 (72.7)	0 (0)	<0.0001
IGRA, *n* (%)	89 (49.2)	45 (68.2)	44 (38.3)	0.0001
Underlying disease, *n* (%)				
Malignant disease	94 (34.6)	31 (24.2)	63 (43.8)	0.001
Diabetes mellitus	54 (19.9)	24 (18.8)	30 (20.8)	0.67
Transplant recipient	13 (4.8)	8 (6.3)	5 (3.5)	0.28
Liver cirrhosis	12 (4.4)	5 (3.9)	7 (4.9)	0.7
Gastrectomy	7 (2.6)	1 (0.8)	6 (4.2)	0.13
Rheumatoid disease	3 (1.1)	0 (0.0)	3 (2.1)	0.25
HIV infection	1 (0.4)	1 (0.8)	0 (0.0)	0.47

a SD, standard deviation; HIV, human immunodeficiency virus; TB, tuberculosis; IGRA, interferon-gamma release assay.

**FIG 1:**
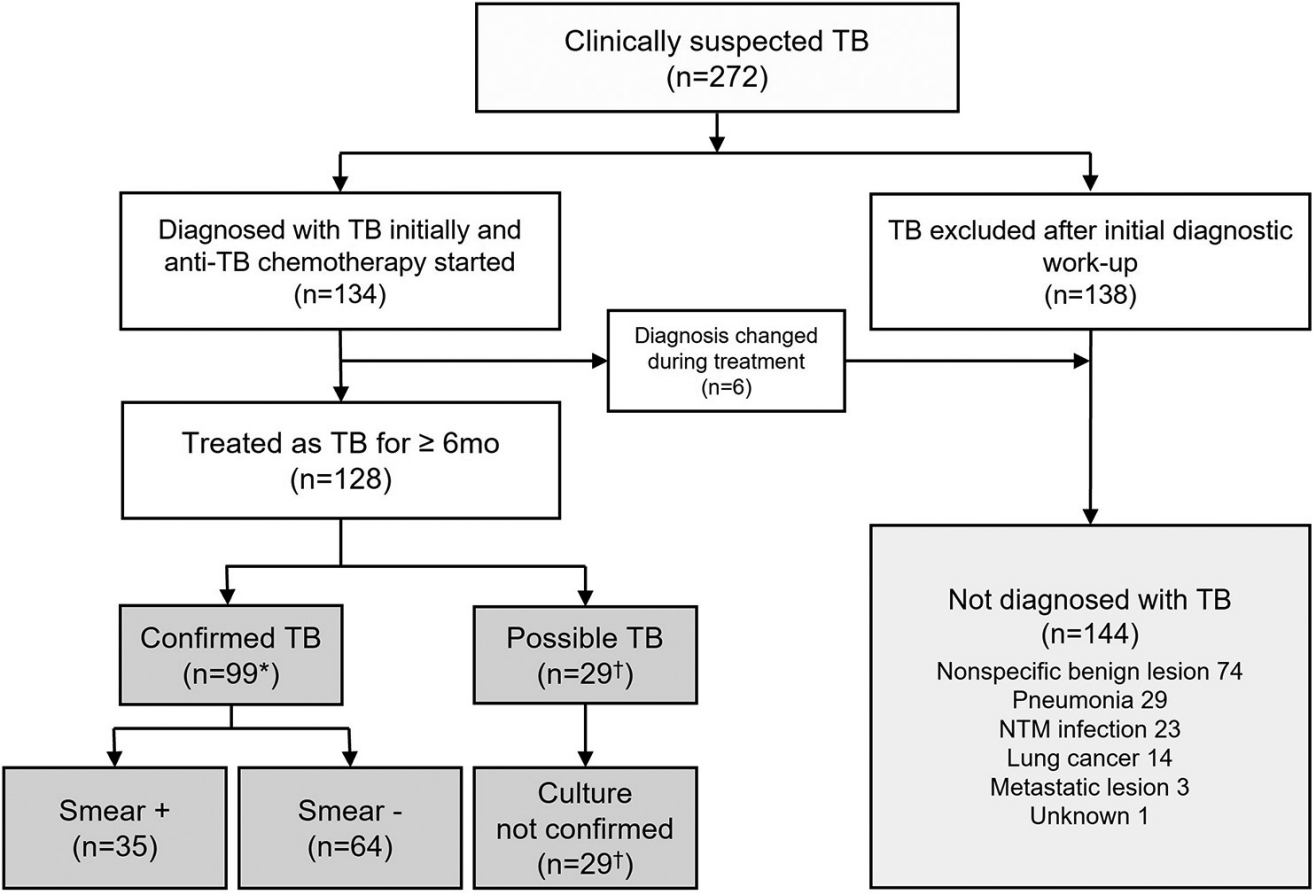
Diagnostic flow of the study patients. Among 272 patients with clinically suspected TB, 128 were finally treated for TB by respiratory or infection specialists who were blinded to the results of the SLIM oral swab assay. Confirmed TB was defined as culture-positive TB patients with at least one positive culture result for MTB from their sputum. Possible TB was defined as culture-negative TB patients with a high clinical likelihood of active TB and a negative mycobacterial culture finding in three or more sputum examinations, but with good clinical and radiographic responses to anti-TB treatment during follow-up without any evidence of an alternative diagnosis. *, six participants with TB not isolated in sputum but isolated from bronchial washing fluid were included. ^†^, one participant who could not produce sputum was included. NTM, nontuberculous Mycobacterium.

A total of 128 patients were finally treated as TB cases and planned to take full-course chemotherapy. They were categorized into smear-positive confirmed TB (*n *= 35), smear-negative confirmed TB (*n *= 64), and possible TB (*n *= 29). Among them, one participant (male, 57 years old) died of an underlying disease (neuroendocrine tumor with liver metastasis) after 3 months of TB treatment and could not complete the treatment. However, he was grouped as smear-negative culture-negative clinical TB because he had a good and persistent response to the TB treatment. The remaining 144 patients did not meet the criteria of TB diagnosis according to the study definition (Fig. 1).

### Clinical validity of the assays.

**FIG 2:**
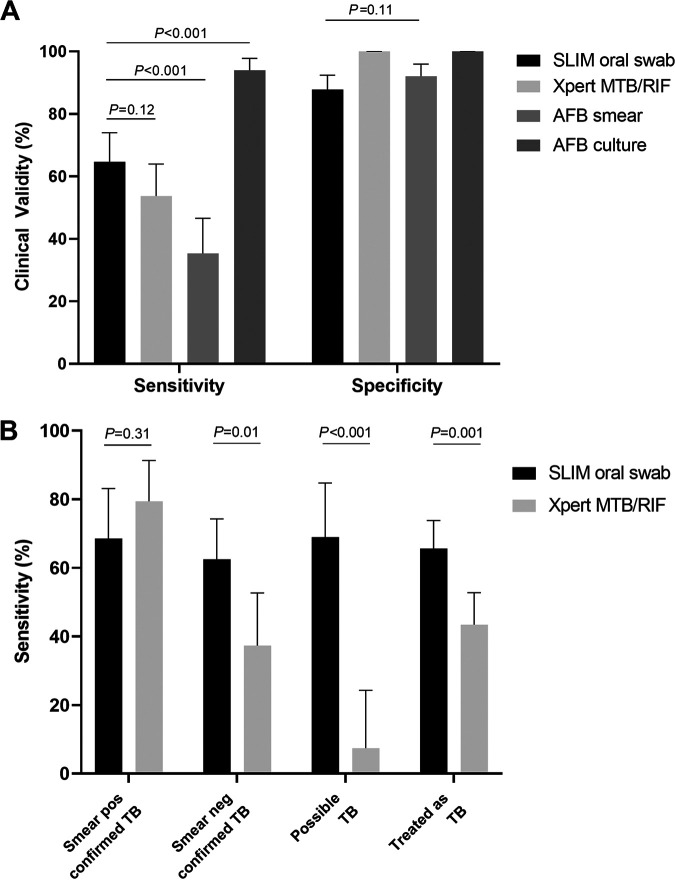
Clinical validity of the SLIM oral swab assay and the Xpert MTB/RIF assay for the diagnosis of TB. (A) Sensitivity and specificity of the five different types of assays for the diagnosis of confirmed TB. (B) Sensitivity of the SLIM oral swab assay and Xpert MTB/RIF according to the categories of TB.

The results of the SLIM oral swab assay and the Xpert assay according to the clinical diagnosis stratified by the AFB smear and MTB culture results are in Table 2, and their diagnostic performances compared with the other tests are in Fig. 2 and Table 3. For confirmed TB, the sensitivity of the SLIM oral swab, Xpert, AFB smear and culture for TB were 64.7% (64/99; 95% CI: 54.4 to 73.8%), 53.7% (51/95; 95% CI: 43.2 to 64.0%), 35.4% (35/99; 95% CI: 26.0 to 46.6%) and 93.9% (93/99; 95% CI: 82.3 to 97.7%), respectively. The sensitivity of the SLIM oral swab assay was higher than that of the Xpert assay, but it did not reach statistical significance (*P* = 0.12). The specificity of the SLIM oral swab, Xpert, AFB smear, and MTB culture were 86.1% (124/144; 95% CI: 86.1 to 91.3%), 100% (130/130; 95% CI: 97.2 to 100.0%), 92.0% (127/138; 95% CI: 86.2 to 96.0%), and 100% (138/138; 95% CI: 97.4 to 100.0%), respectively.

**TABLE 2:** Comparison of sputum exam results according to the categories of TB

	SLIM oral swab (*n *= 272)			Xpert MTB/RIF (*n *= 252^*a*^)		
Case definition	Positive	Negative	Total	Positive	Negative	Total
Confirmed TB						
Smear positive	24	11	35	27	7	34
Smear negative	40	24	64	24	37	61
Possible TB						
Culture negative	20^*b*^	9	29	2	25	27
Not TB	20^*c*^	124	144	0	130	130

a The results of Xpert MTB/RIF were not available for 20 patients.

b One patient without a sputum exam was included. See Fig. 2 for more details.

c See Table S1 for more details. Xpert MTB/RIF was not available for one participant.

**TABLE 3:** Diagnostic performance of the TB assays according to the categories of TB

Case definition	Sensitivity % (n/N, 95% CI)	Specificity % (n/N, 95% CI)	PPV^*a*^ % (n/N, 95% CI)	NPV % (n/N, 95% CI)	Positive likelihood Ratio (95% CI)	Negative likelihood Ratio (95% CI)
Confirmed TB (*n *= 99^*b*^)versus not TB (*n *= 144)						
SLIM oral swab	65 (64/99, 54–74)	86 (124/144, 79–91)	76 (64/84, 68–83)	78 (124/159, 73–82)	4.66 (3.02–7.17)	0.41 (0.31–0.54)
Xpert MTB/RIF	54 (51/95, 43–64)	100 (130/130, 97–100)	100 (51/51)	75 (130/174, 70–79)	Not applicable	0.46 (0.37–0.58)
AFB smear	35 (35/99, 26–46)	92 (127/138, 86–96)	76 (35/46, 63–86)	66 (127/191, 63–70)	4.44 (2.37–8.30)	0.70 (0.60–0.82)
MTB culture	94 (93/99, 87–98)	100 (138/138, 97–100)	100 (93/93)	96 (138/144, 91–98)	Not applicable	0.06 (0.03–0.13)
Possible TB (*n *= 29) versus not TB (*n *= 144)						
SLIM oral swab	69 (20/29, 49–85)	86 (124/144, 79–91)	50 (20/40, 38–62)	93 (124/133, 89–96)	4.978 (3.09–7.98)	0.36 (0.21–0.62)
Xpert MTB/RIF	7 (2/27, 1–24)	100 (130/130, 97–100)	100 (2/2)	84 (130/155, 82–85)	Not applicable	0.93 (0.83–1.03)
AFB smear	0 (0/28^*c*^, 0–12)	92 (127/138, 86–96)	0 (0/11)	82 (127/155, 81–83)	0.00	1.09 (1.04–1.14)
MTB culture	0 (0/28^*c*^, 0–12)	100 (138/138, 97–100)	Not applicable	83 (138/166, 83–83)	Not applicable	1.00 (1.00–1.00)

a PPV, positive predictive value; NPV, negative predictive value; CI, confidence interval; AFB, acid-fast bacilli; MTB, Mycobacterium tuberculosis.

b Six participants with TB not isolated in sputum but isolated from bronchial washing fluid were included.

c One participant without a sputum exam was included. See Fig. 2 for more details.

The sensitivity of the SLIM oral swab assay and the Xpert assay were further analyzed according to the four categories of TB: smear-positive confirmed, smear-negative confirmed, possible, and treated as TB (Fig. 2B). The sensitivity of the SLIM oral swab assay was not significantly different according to the TB categories and ranged from 62.5% to 69.0% (*P* = 0.55). In contrast, the sensitivity of the Xpert assay was the highest in smear-positive confirmed TB (79.4%) and was significantly lower in smear-negative confirmed TB (37.3%, *P* = 0.0002) and possible TB (7.4%, *P < *0.0001). As such, whereas the two assays did not show significant differences in sensitivity for smear-positive PTB (*P* = 0.31), the SLIM oral swab assay had significantly higher sensitivity than the Xpert assay in both smear-negative confirmed TB (*P* = 0.01) and possible TB (*P < *0.0001). The sensitivity of the SLIM oral swab relative to Xpert was significantly superior to that of Xpert relative to the SLIM oral swab (*P* = 0.039, Table S1).

A combination of the SLIM oral swab assay and Xpert assay was evaluated for its clinical usefulness. The SLIM oral swab found 31 additional patients with confirmed TB and 18 with possible TB. Accordingly, this combination showed a sensitivity of 86.3% (95% CI: 77.7% to 92.5%) and specificity of 85.4% (95% CI: 78.1% to 91.0%; Table S2).

### Clinical characteristics of the patients with false-positive results on SLIM oral swab.

A total of 20 patients with positive SLIM oral swab results and the presence of MTB DNA confirmed by Sanger sequencing were not finally diagnosed with PTB according to the study definition. Among them, 10 (50.0%) patients had an inflammatory scar on the chest CT suspected of old inactive TB, and six of them had a history of TB treatment. Another five (25.0%) patients had lesions suspected of active PTB. However, they were regularly followed up without treatment because the physician considered that the clinical evidence for treatment was not sufficient. Three (15.0%) patients had pneumonic infiltration and were treated with broad-spectrum antibiotics, and the other two (10.0%) were diagnosed with NTM pulmonary disease. The detailed characteristics and representative chest images of these patients are provided in the online supplement (Table S3 and Fig. S2).

## DISCUSSION

In this real-world practice setting study, we showed that the SLIM oral swab assay, a non-sputum-based diagnostic test, can detect PTB with high sensitivity, comparable to conventional sputum-based tests, such as mycobacterial culture and Xpert. The superiority of the SLIM assay was particularly pronounced for smear-negative PTB, especially culture-negative clinical PTB, which are cases with a low bacterial load.

The development of a rapid, accessible, and highly sensitive diagnostic tool is a major challenge in the control and management of TB. Among a total of 10 million new TB cases worldwide in 2019, as many as 2.9 million cases were estimated to have been not diagnosed or detected (1), which may be the main source of its transmission and morbidity. As an effort to overcome this diagnostic gap, the WHO recommended the use of the Xpert assay as the initial test for TB (11). However, the sensitivity of the Xpert assay is not high enough for paucibacillary TB cases (4, 12), and is thus limited for use in smear-negative TB cases that require more sensitive diagnostic methods. To meet this unmet clinical need, we applied the SLIM assay, a new generation pathogen enriching technique, and demonstrated its efficacy in PTB and other infectious diseases (9, 10, 13).

In conventional assays for DNA extraction, only a small volume (between 100 μL and 200 μL) of clinical samples is used for the detection of pathogens due to the capacity of the assays; in contrast, the SLIM assay can use both small volumes (between 100 μL and 1 mL) and large volume (more than 1 mL) samples by enabling simultaneous concentration and extraction of the pathogens in a single system. Due to this advantage, the sensitivity of the SLIM system for pathogen diagnosis is significantly higher than that of conventional assays (9, 10, 13).

In our previous study, SLIM assays with 2 mL sputum had a higher sensitivity than the Xpert assay for the diagnosis of culture-positive pulmonary TB (57% [95% CI: 39% to 73%] Xpert versus 91% [95% CI: 78% to 97%]) (SLIM 2 mL) (10). In addition, the SLIM system can minimize the time (<50 min for pathogen enrichment and DNA extraction), cost ($5 to $6), instrument requirements (centrifuges and vortexes), and additional reagents (antibodies) for sample processing considering the material required (9, 10).

Easier, safer, and more effective sampling methods are essential in TB diagnosis (14). Many patients struggle to produce an adequate amount of sputum for testing. Non-sputum-based samples, such as saliva, urine, blood, and exhaled breath concentrate, have been tested, but these samples are typically less useful than sputum (15–17). Recent studies have suggested the use of oral swab samples, which can easily be obtained through noninvasive, non-aerosol-producing methods. Previous studies have shown that MTB DNA can be detected in oral swabs from human and nonhuman primates (18–20). Wood et al. (21) reported 90% sensitivity of oral swab samples, although the number of participants was small, and more than half of them (60%) were smear-positive. Luabeya et al. (22) reported that oral swab samples had 92.8% sensitivity and 91.5% specificity. These two studies showed promising results, but both used two swabs instead of one and included participants with chronic respiratory symptoms, which might increase the sensitivity of those who could be distinguished as TB.

In our study, the SLIM assay was applied to detect PTB using a single oral swab sample per patient suspected of TB. This new method overcame the main limitation of the currently available diagnostics. It increased the sensitivity, which is relatively low in Xpert in cases of smear-negative PTB, and it showed the potential usefulness of non-sputum-based assays, whose efficacies are comparable or even superior to conventional sputum-based techniques. Non-sputum-based assays will be especially helpful in mass-screening large groups of people (e.g., school, prison, military base), in which obtaining sputum may be difficult, such as from children or those without symptoms (23, 24). It will also be useful in the setting of a TB outbreak investigation, in which many asymptomatic active cases may be present (25). The non-sputum-based sensitive examination will reduce the unnecessary spread of sputum production during mass screening by preselecting those few individuals who need a sputum exam.

There is also an unmet clinical need for diagnosing extrapulmonary TB and PTB in patients who cannot produce an adequate amount of sputum. The sensitivity of Xpert in the diagnosis of PTB is very low when using samples other than sputa, such as exhaled breath condensate (0%) and saliva (38.5%) (15). For other clinical samples, such as pleural fluid from TB pleurisy, the sensitivity was quite low at 30% (26). Other techniques showed the possibility of the detection of MTB DNA from plasma (27). Thus, further validation of the SLIM assay with various types of clinical samples and additional development of the system with automation can widen its clinical utility. We expect that our ongoing studies on the application of the SLIM assay to various specimens, such as cerebrospinal fluid (CSF), blood, and oral swabs will be able to provide useful data on this issue. These strengths, its high sensitivity, and application to various samples indicate the future direction and potential uses of the SLIM assay in TB diagnosis. The SLIM assay can increase the sensitivity and have a validated specificity by integrating with the Xpert platform. It can also be used as a rapid diagnostic for TB suspected patients without sputum. A more simplified method with automation will be a critical step to increase its utility independent of an extensive laboratory facility.

This study and the SLIM assay still have some limitations to overcome. First, the false-positive results should be further controlled. The reasons for this were not determined in this study. It may be related to the high sensitivity of the new method, the detection of remnant bacilli or DNA from previous infections, contamination of samples during collection or analysis, or combinations of these things. Further work is needed to discern these possibilities. As Xpert is a closed cartridge diagnostic system that uses automated PCR testing to detect TB with high specificity, the development of a closed SLIM assay may be a key factor in increasing the specificity of the SLIM assay for TB. The development of the automated SLIM system would reduce the problems that result from uncontrollable contaminants by minimizing the external exposure of the sample. Because the microfluidic chip of the SLIM assay is small and requires only an input system that can inject samples, buffers, and air, it can be easily combined with an automated detection system, such as Xpert, which does not have a pathogen concentration system, and it would be able to compensate for the shortcomings of low sensitivity.

Second, a comparison with the Xpert MTB/RIF Ultra assay, which was not available during the initiation step of the study period, should be carried out. Third, the sampling techniques for oral swabs were not compared because this study was targeted to examine the clinical utility of using a single sample in a real practice setting. The swabbing sites, times, and collection kit can affect the results (28, 29), and further study can elucidate if these factors affect the results, especially in this assay designed to increase the sensitivity.

Fourth, the sensitivity of Xpert in this study was relatively low compared with other studies (30, 31). The inclusion of a small number of smear-positive patients and many asymptomatic patients (49.3%) might be the reason. This study was performed in a metropolitan city where medical facilities are easy to access, and regular health checkup services are quite active. Therefore, this study setting makes it difficult to extrapolate our findings to countries with a high burden of TB, such as South Africa. The disease prevalence can affect the positive or negative predictive value (32). The utility of diagnostics should be considered in various clinical and geographical settings.

In conclusion, the sensitivity of the oral swab-based SLIM assay for the diagnosis of PTB was comparable to that of conventional sputum-based methods. The superiority of the SLIM assay in terms of sensitivity was more pronounced in cases of smear-negative PTB, for which tests with a higher sensitivity are critically needed. Further studies on the application of automation and the reduction of false-positive results will expand the role of the SLIM assay in various clinical settings by using both sputum and non-sputum-based samples.

## MATERIALS AND METHODS

### Participants.

Adult patients (>18 years of age) who were clinically suspected of active PTB were prospectively enrolled in two tertiary university-affiliated hospitals in Seoul, Republic of Korea (Asan Medical Center and Severance Hospital) from May 2019 to October 2020. The suspicion of PTB was based on the participants’ symptoms, history, and radiographic findings suggestive of TB (33, 34), and the enrollment was decided by three respiratory and infection specialists (SHK, YAK, and SWL) who each had experience in TB treatment for more than 15 years. Patients who could not understand the study design or the instructions for the sputum exam were excluded.

### Study design and case definition.

After receiving informed consent from each patient and before starting the treatment, two trained researchers (YC and YAK) performed oral swabs using the OMNIgene.ORAL OMR-110 kit (DNA Genotek, Ottawa, Canada) according to the manufacturer’s instructions. In brief, the swabs were brushed in a back-and-forth motion along the participants’ palate, upper gum line, and tongue dorsum for about 10 s (5 to 6 times for each site, a total of 20 times), taking care not to reach back into the mouth. The swab was then inserted into a tube containing stabilizing liquid and the samples were immediately sent to the laboratory and kept at −80°C until analysis. All other steps were performed according to the routine practice of clinically suspected TB for these enrolled participants. Acid-fast bacilli (AFB) smears and mycobacterial cultures (both liquid and solid culture) were examined at least two times and the Xpert assay was performed according to the routine practice. AFB smear and cultures were examined by fluorochrome staining using auramine-rhodamine and culturing in a 3% Ogawa medium and mycobacteria growth-indicator tube medium (MGIT; Becton Dickson, NJ, USA) (34). If the patients could not produce a sufficient sputum sample, sputum induction with 3% normal saline nebulization was performed. The sputum samples were collected after taking the oral swab samples at the same visit from each patient.

TB cases were defined as those treated with anti-TB chemotherapy for at least 6 months according to the American Thoracic Society (ATS), Infectious Disease Society of America (IDSA), and Korean guidelines at the discretion of the respiratory and infectious specialists (SHK, YAK, and SWL) (34, 35) and the fulfillment of full-term treatment. ‘Confirmed TB’ was defined as culture-positive TB patients with at least one positive culture result for MTB from their sputum. Confirmed TB patients were considered smear-positive if they had at least one positive smear result (inclusive of scanty positive smears). ‘Possible TB’ was defined as culture-negative TB patients with a high clinical likelihood of active TB and good clinical and radiographic responses to anti-TB treatment during follow-up without any evidence of an alternative diagnosis, but the mycobacterial culture was not confirmed at the initial and follow-up sputum examinations. The specialists who decided on the TB treatments were blinded to the results of the SLIM assay. We intended to enroll more than 270 participants according to the estimation of the difference in sensitivity of 0.15 (36), a variance of 0.35, 95% CI, desired power of 0.8, and exclusion of 10% in the final analysis. The institutional review boards of Asan Medical Center (2018-0020) and Severance Hospital (4-2018-0029) approved this study, and the protocol of this study was registered at clinicaltrials.gov (NCT03423550).

### Oral sample analysis.

Oral swabs were used for the SLIM assay (SLIM oral swab), and Fig. S1 depicts the overall workflow of the SLIM assay. The principle and the detailed structure of the SLIM assay have been described previously (9, 10, 13, 37). Briefly, the SLIM assay is based on a combination of a microfluidic platform with low-cost thin film and homobifunctional imidoesters (HIs) reagents for MTB enrichment and DNA extraction from the oral swab samples. HIs have two imido ester groups and act as cross-linkers. The imido ester groups of HI form an amidine bond with an amine group on the surface of the thin film. The remaining imido ester groups enable enrichment of MTB cells and extraction of MTB DNA based on electrostatic interactions with negatively charged MTB cells and DNA due to the positive charge of HIs and covalent bonding with the amine group of fragmented DNA due to the imido ester groups of HIs. The detailed processing techniques were described in the online supplement and all experiments were performed by a researcher (BK) blinded to the final diagnosis.

The extracted DNA was used to detect the IS*6110* transposase and catalase-peroxidase (*KatG*) gene. The amplified DNA product from conventional PCR was purified, and TB was confirmed using Sanger sequencing. Diagnosis of TB was carried out according to the schematic flow. All conventional PCR (endpoint PCR) reactions were performed using the extracted DNA as a template from 272 oral swab samples, and all results were reported as “positive” or “negative” to determine TB (Fig. S3 and S4). Based on these results, the sensitivity and specificity were calculated as described in the next section.

### Statistical analysis.

Baseline characteristics were compared by Student's *t* test for continuous variables or chi-square tests for categorical variables. To compare the clinical validity between tests, McNemar’s chi-square test was used. All data are expressed as mean ± standard deviation unless noted otherwise. All statistical analyses were performed using IBM SPSS Statistics for Windows, version 21.0 (IBM Corp., Armonk, NY, USA).

### Data availability.

Individual participant data collected during the study, after deidentification, and the study protocols and statistical analysis code are available beginning 3 months and ending 2 years following article publication to researchers who provide a methodological sound proposal, with approval by an independent review committee. Data are available for analysis to achieve the aims stated in an approved proposal. Proposals should be directed to seiwon@amc.seoul.kr. To gain access, data requestors will need to sign a data access or material transfer agreement approved by Asan Medical Center and Severance Hospital.
