# Supplementary material for: Gene-Based Diagnosis of Tuberculosis from Oral Swabs with a New Generation Pathogen Enrichment Technique
Source: Microbiol Spectr. 2022 May 19;10(3):e00207-22. doi: 10.1128/spectrum.00207-22 (PMC9241603; doi:10.1128/spectrum.00207-22)
Supplement: SUPPLEMENTAL FILE 1 — Supplemental material. Download spectrum.00207-22-s001.pdf, PDF file, 0.6 MB [file spectrum.00207-22-s001.pdf]

Online Supplement

Gene-based Diagnosis of TB from Oral Swab with a New Generation Pathogen Enrichment Technique

Young Ae Kang, Bonhan Koo, Ock-Hwa Kim, Joung Ha Park, Ho Cheol Kim, Hyo Joo Lee, Myoung Gyu Kim, Youngwon Jang, Yong Seo Koo, Na Hyun Kim, Yong Shin, Sei Won Lee and Sung-Han Kim

Online Supplementary Materials

Detailed Methods

Supplementary Table

Supplementary Figure Legend

Reference List for Online Supplementary Materials

Detailed Methods

Oral sample analysis

Oral swabs were used for the SLIM assay (SLIM oral swab), and Figure 1 depicts the overall workflow of the SLIM assay. The principle and the detailed structure of the SLIM assay have been described previously (1-4). Briefly, the SLIM assay was based on a combination of a microfluidic platform with low-cost thin film and homobifunctional imidoesters (HIs) reagents for MTB enrichment and DNA extraction from the oral swab samples. HIs can be captured from the surface of MTB cells by electrostatic coupling and they bind with DNA by electrostatic coupling and covalent binding. The oral swab samples (1 mL) were liquefied with liquefaction solution (4% NALC, 1·45% sodium citrate, and 2·67% NaOH) at a 1:1 ratio as according to the method described by Ganoza et al (5). The liquefied oral swab samples (2 mL) were mixed with dimethyl pimelimidate (DMP), one of the HIs (100 mg/mL), and injected into the SLIM platform using a syringe pump after a modification step with O_2 plasma and 3-aminopropyl diethoxymethylsilane. After incubation at room temperature, the debris was removed and the cells were lysed by incubation with lysis buffer with proteinase K and DMP solution at 56 °C. To collect the DNA from the oral swab samples, 100 μL of elution buffer was injected into the SLIM platform after washing with 1 mL of PBS.

For the detection of MTB obtained from the oral samples, we used conventional *Mycobacterium tuberculosis* PCR to detect the IS*6110* transposase and catalase-peroxidase (*KatG*) gene of TB using the Taq PCR Core Kit (Qiagen, Hilden, Germany). Further PCR-based confirmation was carried out by the amplification of the 308-bp DNA fragment specific for the IS*6110* gene using IS*6110*_F (5′- ACGGTTCAGGGTTAGCCACA -3′) and IS*6110*_R (5′-TTAAAGACCGCGTCGGCTTTC -3′) primers and the 555-bp DNA fragment specific

for *KatG* gene using *KatG*_F (5′-AACTCGTCGGCCAATTCCTC-3′) and *KatG*_R (5′-GCAGATGGGGCTGATCTACG-3′) primers in all clinical isolates. The PCR process consisted of an initial denaturation step at 95°C for 15 min; 45 cycles of 95°C for 30 s, 60°C for 30 s, and 72°C for 30 s; and a final elongation step at 72°C for 10 min. DNA (5 μL) was amplified in a total volume of 25 μL containing 10× PCR buffer, 2·5 mM MgCl, 0.25 mM deoxynucleotide triphosphate, 25 pmol of each primer, and 1 unit of Taq DNA polymerase. Gel electrophoresis was performed to separate the PCR products on a 2% agarose gel containing LoadingSTAR (A750, Dyne Bio Inc., Seoul, Republic of Korea). The gel was visualized using the ChemiDoc XRS+ system (Bio-Rad, Marnes-la-Coquette, France). All results were reported as “positive” or “negative”.

Table S1. Sensitivity and specificity of SLIM oral swab and Xpert relative to each assay and AFB culture

A. Diagnostic accuracy of SLIM oral swab as the standard reference of Xpert

Reference standard	SLIM oral swab	
	Sensitivity	Specificity
Xpert	30/54 (55.6%)*	114/133 (85.7%)*
Mycobacterial culture	61/93 (65.6%)	118/138 (85.5%)

B. Diagnostic accuracy of Xpert as the standard reference of SLIM oral swab

Reference standard	Xpert MTB/RIF	
	Sensitivity	Specificity
SLIM oral swab	30/80 (37.5%)†	111/111 (100%)†
Mycobacterial culture	50/90 (55.6%)	130/130 (100%)

The sensitivity of SLIM oral swab relative to Xpert is significantly superior to that of Xpert relative to SLIM oral swab (p=0.039).

Table S2. The concordance analysis of two assays

SLIM oral swab	Xpert MTB/RIF	Confirmed TB		Possible TB	Not TB
		Smear positive	Smear negative		
(+)	(+)	18	11	1	0
(+)	(–)	5	26	18	19
(–)	(+)	9	13	1	0
(–)	(–)	2	11	7	111
Total		34	61	27	130

Table S3. Characteristics of the patients with a false-positive result in the SLIM oral swab assay

No.	Sex	Age	Xpert MTB/RIF	AFB smear	AFB culture	QFT-plus	Clinical situation
1	M	36	Negative	Negative	No growth	Positive	Bilateral centrilobular nodules on CT, improved with antibiotics
2	F	69	Negative	Negative	No growth	Negative	RUL infiltration on regular follow-up
3	M	58	Negative	Negative	No growth	Positive	RUL infiltration, anti-TB chemotherapy for 2 months, stopped due to side effects
4	M	56	Negative	Negative	No growth	Positive	RUL infiltration on regular follow-up
5	F	60	Negative	Negative	No growth	Positive	LUL infiltration, treated as latent TB infection
6	M	59	Negative	Negative	No growth	Positive	LUL infiltration on regular follow-up, lesion slightly increased
7	F	72		Negative	No growth	Positive	RUL inflammatory scar on regular follow-up
8	F	65	Negative	Positive	*M. avium*	Negative	Treated as NTM pulmonary disease
9	M	60	Negative	Negative	No growth		RUL inflammatory scar on regular follow-up
10	M	66	Negative	Negative	*M. intracellurare*		Followed up as NTM pulmonary disease without treatment
11	F	54	Negative	Negative	No growth		RUL inflammatory scar on regular follow-up
12	M	76	Negative	Negative	No growth		RUL infiltration on regular follow-up
13	M	57	Negative	Negative	No growth		BUL inflammatory scar on regular follow-up
14	F	68	Negative	Negative	No growth	Positive	RUL infiltration on regular follow-up
15	M	41	Negative	Negative	No growth	Positive	LUL inflammatory scar on regular follow-up
16	M	76	Negative	Negative	No growth		RUL inflammatory scar on regular follow-up
17	F	45	Negative	Positive	*M. intracellurare*	Negative	Treated as NTM pulmonary disease
18	F	64	Negative	Negative	*M. intracellurare*		Followed up as NTM pulmonary disease without treatment
19	M	77	Negative	Negative	No growth		LUL infiltration, TB relapse suspected but lesion slightly improved without treatment
20	F	56	Negative	Negative	No growth		Bilateral centrilobular nodules on CT, improved with antibiotics

QFT-plus, QuantiFERON-TB Gold Plus; RUL, right upper lobe; LUL, left upper lobe; TB, tuberculosis; NTM, non-tuberculous mycobacterium; BUL, both upper lobe.

Blank cells indicate that the test was not performed.

Figure legends

Figure S1. Schematic representation of the workflow of the SLIM assay for the tuberculosis diagnosis.

A mixture of oral swab sample and liquefaction solution with HI reagent is added to the SLIM system to enrich the *Mycobacterium tuberculosis* (MTB) and extract the DNA without the need for detergents or bulky instruments. (1) After injecting 2 ml of liquefied oral swab samples into the SLIM system, the negative charged MTB binds to the positively charged HI for enrichment. (2) Then, DNA from the enriched MTB is isolated by the covalent bonding with HI. (3) After washing with PBS, the DNA is eluted for MTB detection with either PCR or real-time PCR.

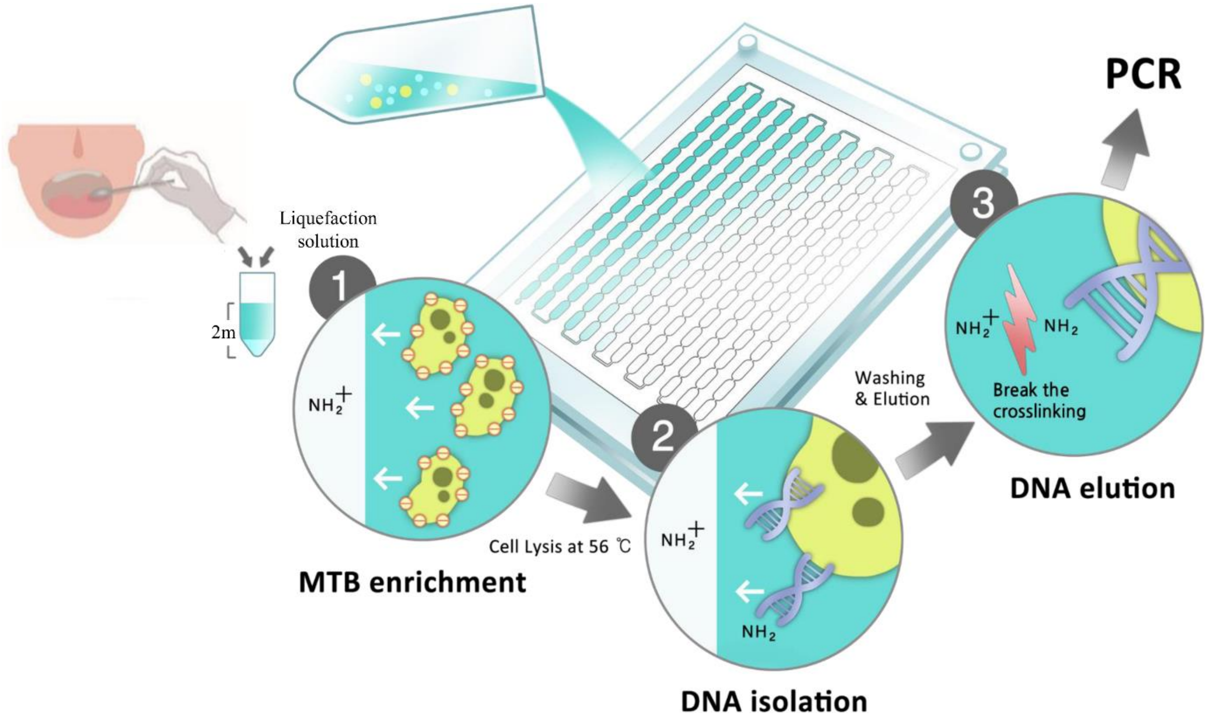

Figure S2. Representative chest images of a patient with a false-positive result on the SLIM oral swab assay.

(**A**) A 69-year-old woman (patient #2 in Table S1) visited our clinic for an incidentally found right upper lobe infiltration on a routine exam without respiratory symptoms. Pulmonary TB was strongly suspected due to the slowly progressing inflammatory lesion in the upper lobe, but her sputum exam did not show any causative organism and she was reluctant to take anti-TB chemotherapy without definite evidence. (**B**) After one year, her lesion was slightly improved without treatment.

(**C**) A 58-year-old man (patient #3 in Table S1) was referred for an incidentally found infiltration in the right upper lobe during work-up for hepatocellular carcinoma and liver transplantation. Based on the positive result in the interferon-gamma releasing assay (QuantiFERON-TB Gold Plus [QFT-Plus]), a treatment for active TB or latent TB infection was necessary before transplantation. The treatment for active TB was initiated and the patient initially showed responses to the treatment; however, the treatment was stopped after 3 months due to side effects and a negative result in AFB culture.

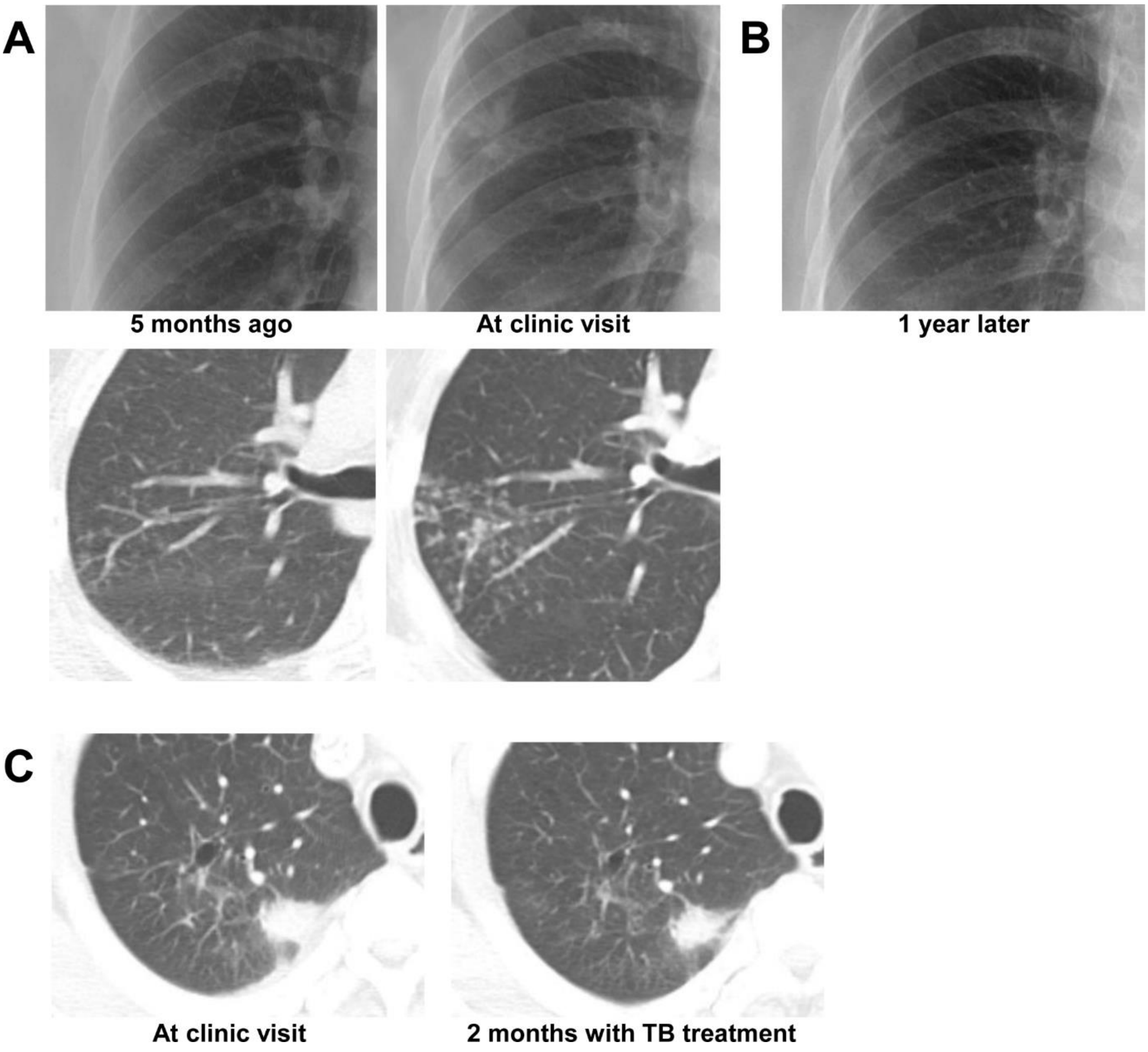
A
B
5 months ago
At clinic visit
1 year later
C
At clinic visit
2 months with TB treatment

Figure S3. Schematic work-flow of *Mycobacterium tuberculosis* PCR in SLIM assay.

Diagnosis of TB was carried out according to the schematic work-flow. DNAs were extracted from 272 oral swab samples, including confirmed TB, possible TB, and not TB, using SLIM assay. The amplified DNAs with the specific primers of the *IS6110 transposase* and *catalase-peroxidase* (*KatG*) genes were analyzed by using agarose gel and Sanger sequencing methods. In the 1st PCR, 272 oral swab samples were classified as Positive (two gene positives), Negative (two gene negatives), and Undecided (one gene positive and one gene negative). Except for 261 confirmed results, 2nd PCR was performed with 11 undecided samples from the 1st PCR. Then, 3rd PCR was performed with one undecided sample from the 2nd PCR. As a result, 272 samples were classified as 104 positives and 144 negatives.

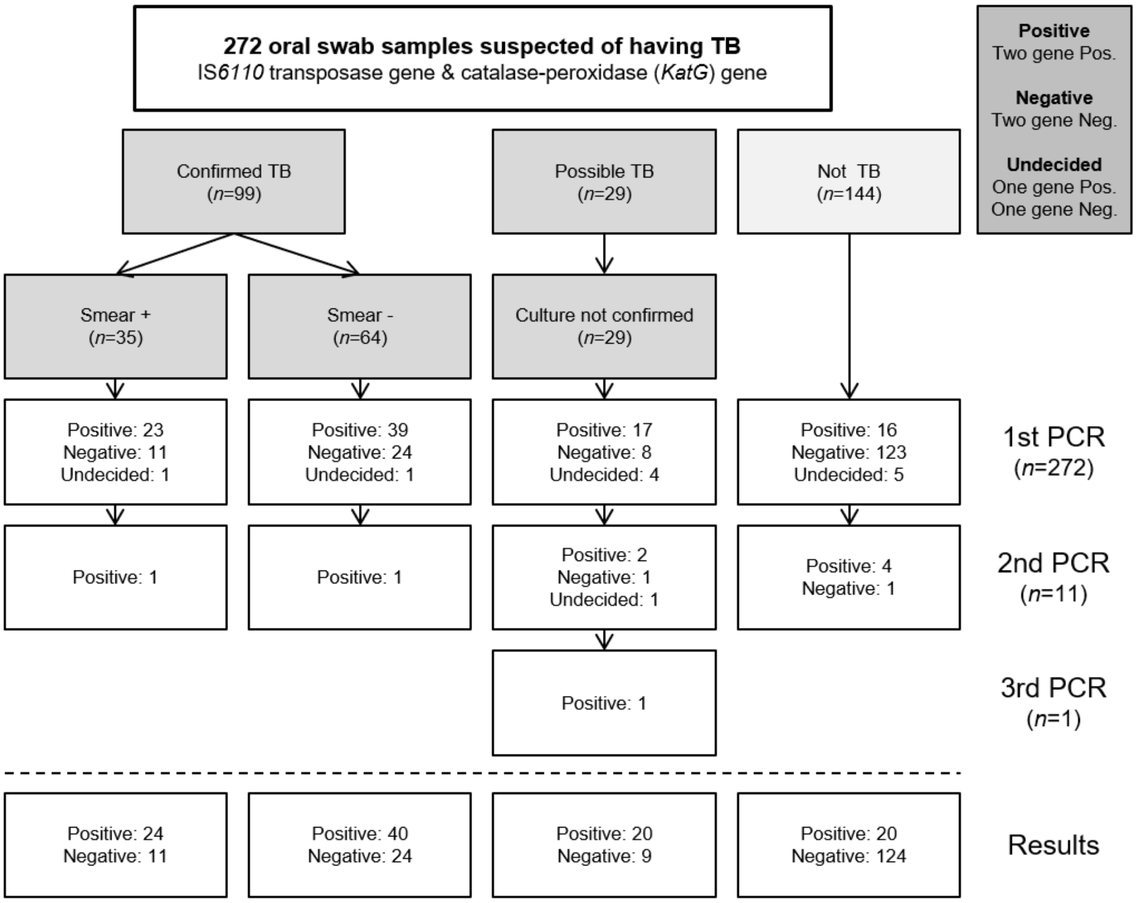

Figure S4. Confirmation of positive results for TB detection in the SLIM assay.

The extracted DNA was used to detect the *IS6110 transposase* and *catalase-peroxidase (KatG)* gene by end-point PCR assay. Then, the amplified DNA product was purified and TB was confirmed using Sanger sequencing and Basic Local Alighment Search Tool (BLAST) system from National Center for Biotechnology Information (NCBI).

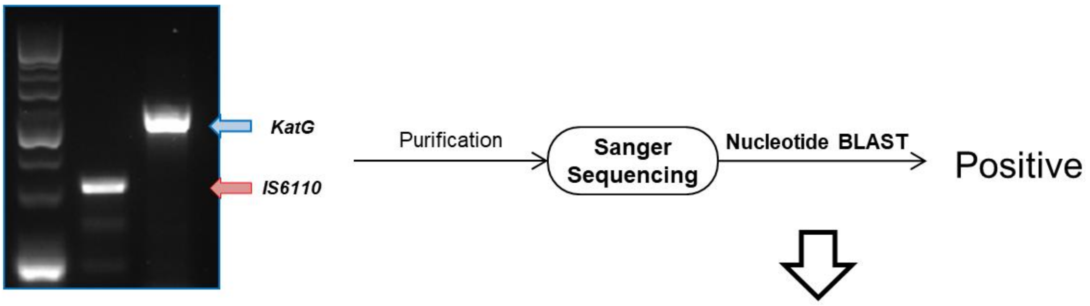

Sequencing using *IS6110* primer

100 sequences selected

Organism	Blast Name	Score	Number of Hits	Description
Mycobacterium tuberculosis complex	high GC Gram+		100	
. Mycobacterium tuberculosis	high GC Gram+	364	98	Mycobacterium tuberculosis hits
. Mycobacterium tuberculosis variant bovis BCG	high GC Gram+	364	1	Mycobacterium tuberculosis variant bovis BCG hits
. Mycobacterium orygis	high GC Gram+	364	1	Mycobacterium orygis hits

Sequencing using *KatG* primer

100 sequences selected

Organism	Blast Name	Score	Number of Hits	Description
Mycobacterium tuberculosis complex	high GC Gram+		152	
. Mycobacterium tuberculosis	high GC Gram+	462	140	Mycobacterium tuberculosis hits
. Mycobacterium tuberculosis variant bovis	high GC Gram+	462	7	Mycobacterium tuberculosis variant bovis hits
. Mycobacterium tuberculosis variant bovis BCG	high GC Gram+	462	3	Mycobacterium tuberculosis variant bovis BCG hits
. Mycobacterium tuberculosis H37Rv	high GC Gram+	462	1	Mycobacterium tuberculosis H37Rv hits
. Mycobacterium orygis	high GC Gram+	462	1	Mycobacterium orygis hits

References

1. Lee SW, Kang YA, Jin CE, Kim HC, Noh GS, Lee HJ, Park JH, Koo YS, Shin Y, Kim SH. 2020. Gene-based diagnosis of tuberculosis with a new-generation pathogen enrichment technique. Eur Respir J 55:1901885. https://doi.org/10.1183/13993003.01885-2019.
2. Jin CE, Koo B, Lee EY, Kim JY, Kim SH, Shin Y. 2018. Simple and label-free pathogen enrichment via homobifunctional imidoesters using a microfluidic (SLIM) system for ultrasensitive pathogen detection in various clinical specimens. Biosens Bioelectron 111:66-73. https://doi.org/10.1016/j.bios.2018.04.001.
3. Park JH, Jin CE, Koo B, Kwon JS, Cha HH, Kim JY, Noh GS, Koo YS, Jeon SB, Lee SA, Shin Y, Kim SH. 2019. A simple microfluidic assay for diagnosing tuberculous meningitis in HIV-uninfected patients. J Clin Microbiol 57:e01975-01918. https://doi.org/10.1128/JCM.01975-18.
4. Jin CE, Koo B, Lee HJ, Park IJ, Kim SH, Shin Y. 2020. Bis(sulfosuccinimidyl)suberate-based helix-shaped microchannels as enhancers of biomolecule isolation from liquid biopsies. Anal Chem 92:11994-12001. https://doi.org/10.1021/acs.analchem.0c02503.
5. Ganoza CA, Ricaldi JN, Chauca J, Rojas G, Munayco C, Agapito J, Palomino JC, Guerra H. 2008. Novel hypertonic saline-sodium hydroxide (HS-SH) method for decontamination and concentration of sputum samples for *Mycobacterium tuberculosis* microscopy and culture. J Med Microbiol 57:1094-1098. https://doi.org/10.1099/jmm.0.2008/001339-0.
